# Technology Personalities in the Classroom: A New Classification System of TechTypes from Expert to Techy Turtle

**DOI:** 10.3390/pharmacy11030091

**Published:** 2023-05-26

**Authors:** Julie H. Oestreich, Jason W. Guy

**Affiliations:** 1 Department of Pharmaceutical Sciences, College of Pharmacy, University of Findlay, Findlay, OH 45840, USA; 2 Department of Pharmacy Practice, College of Pharmacy, University of Findlay, Findlay, OH 45840, USA

**Keywords:** technology, education, personalities

## Abstract

The incorporation of technology in higher education has increased rapidly in recent years to allow for remote work and to promote active learning. Technology use could align with personality type and adopter status as defined by the diffusion of innovations theory. A review of the literature was conducted using PubMed with 106 articles found, and 2 articles meeting the inclusion criteria of the study. Search terms included “technology AND education”, “pharmacy AND personality”, “technology AND faculty AND personality”, and “technology AND health educators AND personality”. This paper highlights the current literature and introduces a new classification system to describe the technology personalities of instructors. The proposed personality types (TechTypes) include expert, budding guru, adventurer, cautious optimist, and techy turtle. Awareness of the advantages and disadvantages of each personality type—as well as one’s own technology personality—may guide the selection of collaborators and tailor technology training for future growth.

## 1. Introduction

The application of technology expanded rapidly throughout higher education in the early 21st century, and this use was further accelerated during the COVID-19 pandemic [1]. This drastic shift in the communication of information occurred as educators adapted to meet the changing needs and preferences of a new generation of learners [2]. As a result, online programs and hybrid courses have increased in prevalence to allow for more remote and self-guided learning for students. In addition to online learning, technology has expanded the educational options for communication in face-to-face settings through new engagement platforms and tools. 

Importantly, the incorporation of technology provides active learning opportunities for students and improves engagement as well as student learning [3]. Active learning is a required component of the Accreditation Council of Pharmacy Education (ACPE) standards for pharmacy education and helps instructors meet learning objectives in a dynamic way [4]. While methods of incorporating active learning differ among individuals and organizations, all affiliated institutions of ACPE must meet the active learning component. Technology provides one option for meeting these standards, and therefore, a better understanding of its value and how individual instructors can best apply technology using their own skillset and personality may improve the classroom experience and learning. 

One key advantage of technology includes the flexibility to optimize the learning experience for students with different learning styles [5]. Each student possesses a unique learning personality that spans a variety of categories such as visual, auditory, or kinesthetic methods [6]. For example, visual learners—who generally prefer to read and observe rather than talk or act—benefit from technology such as annotation of slides or personal tablets to take notes and draw pictures or figures related to the content [6]. Auditory learners may gain value from music within games and recorded content that allows for these individuals to listen to information multiple times at a slower or faster speed that meets their needs. Likewise, kinesthetic learners can benefit from technology incorporated through more sophisticated hands-on activities such as scavenger hunts or other game-based learning, or simple interactive models in an app or online textbook [6]. Overall, the implementation of technology into the classroom has expanded the learning opportunities for a variety of learning personalities.

Despite its value for learners, increased utilization of technology in the classroom may add stress to instructors [7], particularly if it is implemented rapidly or if tailored training and support are not available. Sufficient resources for faculty training and new technology itself are often needed. Therefore, these financial and labor costs represent a barrier to successful implementation [8]. To enhance the likelihood of success, faculty should also feel prepared—both technically and emotionally—to use the new technology. Indeed, research has shown that self-awareness and technology readiness can improve positive technology outcomes and self-esteem in changing environments [9]. To help faculty become more self-aware and reflective, several tools are available.

Examples of self-assessment resources include Myers–Briggs and CliftonStrengths [10,11,12]. These valuable tools help individuals identify their core traits and values as well as interpret and manage their daily interactions in collaborative teams. Generally, these resources involve a self-assessment process where users answer questions about themselves or choose preferences from different options presented. After completing the assessment, the individual receives results, which could include a personality type or a list of strengths for the user. While these resources are useful for identifying core skills and characteristics, they reveal less about an instructor’s personality in the classroom and do not emphasize technology. Personality tests also do not generally predict how likely an individual is to adopt technology into the classroom. Therefore, a technology personality assessment specific to education may facilitate the usage of technology in the classroom and reveal strengths and areas of growth for individual instructors.

The goal of this paper was to review the current literature on technology personalities in education and to create a new classification of relevant technology personalities called TechTypes for self-reflection and application. 

## 2. Overview of the Literature

A review of the literature was conducted utilizing PubMed and the MeSH search terms “technology AND education”, “pharmacy AND personality”, “technology AND faculty AND personality”, and “technology AND health educators AND personality”. The searches yielded 106 article results. Inclusion criteria required the article to focus on the personality types of educators. Titles and abstracts were scanned to ensure that articles met criteria for inclusion and relevance. Articles focused on technology in general or new uses for technology without mention of the user were excluded from consideration. Duplicate studies were removed, and poster presentations were not captured. 

## 3. Results

A review of the literature during the winter of 2022 found minimal information on the different technology personality types of educators. The PubMed search identified 106 article results; however, only two were relevant to the topic of technology personalities in education [8,9]. While scant evidence exists regarding technology personality types (see Table 1), ample literature describes a related concept called the diffusion of innovation theory—first noted by Everett Rogers—which explains the adopter status of new innovations [8,13,14,15,16]. The diffusion of innovation theory suggests that a spectrum exists among individuals based on the likelihood to adopt new technology [15]. This theory groups people based on their likelihood of implementing a change or innovation [15]. These groupings range from innovators and early adopters to late majority and laggards based on personality characteristics (refer to Table 2). Beyond this theory, evidence suggests that less experienced faculty tend to have a more positive attitude for increased technology usage and adoption [17], although an individualized assessment is preferred to generalizations.

When applied specifically to the educational setting, the diffusion of innovation theory would separate faculty into groups—innovators, early adopters, early majority, late majority, and laggards—based on their speed to incorporate new technology (refer to Table 2) [8,15,16]. This speed of adoption is important as evidence suggests that making the transition from early adopters to the early majority critically determines the likelihood that a technological innovation will successfully integrate into the educational setting [8]. Failure to bridge this gap may leave the innovation isolated to the small number of innovators and early adopters, while failing to expand to the vast majority of faculty in the early and late majority [8]. Collectively, the better understanding of an individual’s comfort with integrating new technology and reflecting on how best to grow in this area offers an opportunity to improve the classroom experience for both students and instructors.

While considering this information, the authors (JHO and JWG) reflected on their experiences as part of a university-wide technology pilot and separate user group designed to explore new technology options at their institution. Through these interactions, the authors recognized vast differences in preferences and strengths for technology implementation with some similarities to the diffusion of innovations model.

Based on the premises of common personality types and the diffusion of innovation theory, the authors proposed five main technology personality types or ‘TechTypes’ for consideration in the academic setting. A TechType is a personality type that could be useful for individuals to identify with and to help them reflect on their strengths and areas for growth. These pilot TechTypes were modeled from the primary literature and the authors’ experiences.

**Expert**

*‘I have easily conquered everything on your list.’*

Expert personality types easily integrate technology into their classroom. These self-starters tackle challenging problems and incorporate new technology rather effortlessly with little support. These individuals have already mastered most common technologies with application to educational settings and routinely explore new technologies. When incorporated into a team, this TechType serves as a vast resource for the institution and peers due to their deep knowledge and advanced skillset. Experts possess similar characteristics to the early adopters of the diffusion of innovation theory. 

**Budding Guru**

*‘I enjoy experimenting with new tech tools in the classroom.’*

Budding gurus—or experts-in-training—enjoy learning from others and adding new technology to their classroom toolkit. These thoughtful and ambitious individuals seek burgeoning opportunities with passion. Along with experts, budding gurus possess a growing knowledge base, and therefore, may serve as effective resources and mentors to train others. Unlike experts, budding gurus may require significant support upfront, similar to the early majority [8,15].

**Adventurer**

*‘I am spontaneous and imaginative, and therefore will try anything.’*

Adventurers—the namesake of the ISFP (Introverted, Observant, Feeling, and Prospecting) personality type—eagerly try new things, and technology is no exception [10]. Adventurers frequently employ innovative and creative approaches. Their free-spirited and curious nature challenges the status quo and helps move technology elements forward. This TechType’s creativity bursts through obstacles, generating fresh ideas and repurposing ideas for new uses across disciplines. This TechType possesses similar characteristics to the innovators of the diffusion of innovation theory.

**Cautious Optimist**

*‘I see potential, but don’t like to take unnecessary technology risks in the classroom.’*

These more guarded individuals often align with the late majority, making sure that new changes are well vetted and that risks are well-balanced by demonstrated benefits. A cautious optimist may also start with a small step to make sure that the integrated technology works well before moving on to full implementation. This technology personality may require significant and sustained support from technology specialists or mentors to maintain momentum through the learning curve of a technology—from novice to confident user.

**Techy Turtle**

*‘I will implement new technology when required or when the benefits clearly exceed my current approach.’*

A techy turtle generally favors reliability and consistency in the classroom and has no interest in pursuing fads or gaining personal recognition for classroom transformations. They often are slower to implement new technology and tend to favor traditional techniques that they know work well for student learning. Not surprisingly, techy turtles typically fall in the late majority or laggard categories for speed of implementation. This technology personality may hinder efforts to standardize new technology as cultural norms in the institution, but also guard from the frivolous implementation of new—and often expensive—tools for the wrong reasons.

Of note, the authors want to emphasize that there is no good/bad or correct/incorrect technology personality. Each of the proposed personality types possess inherent advantages and disadvantages, as highlighted in Table 3. Additionally, the authors provide recommendations to optimize the performance of each personality type.

## 4. Discussion

Although the prior classification of technology adoption based on the classic diffusion of innovations model closely relates to this discussion, our literature search revealed minimal evidence specifically describing the role of technology personalities of instructors. To fill this gap, the authors created a new classification system for the technology personalities of educators, ranging from expert to techy turtle. 

Based on our preliminary experiences, we expect each faculty member will associate with a technology personality type based on their own characteristics and views on technology and pedagogy. The potential value, however, does not lie with a cute placard displaying one’s type, but in the reflection and application of these insights to improve individual and collective technology use. In fact, several of the personality types have distinct advantages that can be amplified by collaborating with a complementary personality type. For example, pairing a cautious optimist with an adventurer can make for an ideal pair, as the authors personally experienced. The adventurer’s (JHO) willingness to try new things can sometimes lead to more risks and incidences of technology failure. However, pairing them with a cautious optimist (JWG in this case) provides a more balanced and measured viewpoint, which helps mitigate unnecessary mishaps. Moreover, the cautious optimist gains the benefit of working with an adventurer to try new ideas never considered or seemed too risky at first. This collaborative model can improve teamwork in the classroom and advance other projects. 

Other collaborative approaches include leveraging experts or budding gurus to serve as mentors for other more risk-averse TechTypes. Serving as a mentor provides an outlet for the expert or budding guru to share their deep knowledge while supplying the cautious optimist or techy turtle with a known peer for beginning their journey with new technology. This familiar support from an expert/budding guru colleague can augment the formal institutional IT support available for any major logistical issues or technological failures. Additionally, this pairing may contribute to effective new collaborations between team members with opposite and complementary skill sets to the benefit of other college or university missions [18]. 

### 4.1. Reflection

The authors propose and describe five different TechTypes and associated characteristics. To date, however, no definitive placement test exists to rank your proclivity for each TechType if none of the descriptions match well. In this case, the authors recommend asking trusted colleagues to consider which TechType matches you best. In many cases, your closest colleagues may identify an accurate selection and provide additional insights on your unique strengths and weaknesses. 

In addition, the authors highly suggest speaking with an IT specialist at your institution to validate your TechType, as well as garner new ideas and build institutional support at the early stages of implementation. These true experts generally manage numerous technology pursuits both within and outside of the work setting and remain well versed in cutting-edge advancements of the technology landscape. Conversations with these professionals may help clarify whether you have mastered most of the burgeoning options (expert), still have plenty more to learn (budding guru), or have never heard of many of the recommendations (cautious optimist or techy turtle).

Finally, if the above suggestions do not work, we recommend reflecting on previous integrations of technology in your courses. Think about what went well and what did not. How did you respond to challenges and progress the use of technology over time? When was the last time you implemented something new? You may be able to match some of the TechType characteristics with your reflection or the feedback provided from close colleagues. 

### 4.2. Other Considerations

The ultimate goal of this discussion is to advance the appropriate and thoughtful use of technology in the classroom to improve student engagement and learning outcomes. Understanding one’s TechType can help initiate the reflective process on new technological changes. Identifying key pros and cons of each TechType as well as recommendations for implementation may prove beneficial for reflecting on strengths and planning new ideas for the future. However, understanding one’s TechType does not mean that improved learning outcomes will immediately follow. The application of this classification system has not been studied, and proportions of each TechType have not been established. In addition, other barriers may play a role in limiting the effect of technology incorporations, such as a perceived lack of benefit and an increase in workload [8]. As mentioned previously, one way to overcome these barriers is through partnerships to maximize benefit and reduce inefficiencies. Recognition of the proposed TechTypes may emphasize the importance of individualized or small group training for increased adoption and user satisfaction. Indeed, developing the time and space for collaborative environments may be useful for instructors reluctant to attend group training sessions not personalized to their skillset or personality [8].

Moreover, faculty should remain thoughtful of when and how they implement technology. The technology must help meet the learning objectives, and not be employed just because it is new or interesting. While technology can improve active learning and engagement, the course cannot lose sight of its focus on the preparation of students through meeting learning objectives and outcomes. Courses with limited use of technology could first identify learning outcomes that may benefit from the integration of technology while keeping the content and learner as first priorities. As highlighted above, creating new collaborative partnerships and accounting for different learning styles may prove useful for generating the best new ideas for maximizing student learning and mitigating common pitfalls. 

Importantly, technology can also improve the efficiency of many processes outside the classroom, and so these other potential benefits should also be considered as part of the broader discussion on appropriate implementation. For example, technological tools may improve communication in the workplace and thereby facilitate goals in faculty research or service to the institution. Furthermore, the continual advancement of technology outside of academia necessitates a discussion of optimal integration in the classroom as many students will pursue careers which rely on expanding technology for primary job duties. Modeling new technology in the classroom prepares students for the workplace where new innovations are commonplace and sometimes employed with little training. 

The authors encourage future research to consider the frequency and barriers to success for each of these personality types in addition to their impact on learning outcomes.

## 5. Conclusions

The authors conducted a literature review that resulted in 106 results; however, few of the articles discussed technology personality types. Most research in this area focuses on the innovative technology itself rather than the personalities of the individuals adopting the technology. Nonetheless, related literature was discovered on the diffusion of innovation theory, and this concept served as a launching point for the TechType discussion. We describe five main TechTypes: expert, budding guru, adventurer, cautious optimist, and techy turtle. Understanding these TechTypes may benefit reflection and the consideration of new innovations in the classroom. While understanding your personality type may provide some value for collaboration and for identifying strengths and weaknesses, the effect on learning outcomes has not been documented and requires further research. Therefore, future aspects of this work could consider whether understanding these roles helps unlock each personality’s full potential and improves student learning through effective technology utilization. 

The key takeaways are as follows:

- This paper introduces five TechTypes including expert, budding guru, adventurer, cautious optimist, and techy turtle.
- Each TechType possesses unique pros and cons, and collaborations between complementary personalities may prove beneficial.
- Understanding one’s TechType may facilitate thoughtful consideration of optimizing technology in the classroom to best meet learning objectives.

## Figures and Tables

**Table 1:** Review of Literature on Technology Personality Types.

Author of Resource	Design	Purpose	Summary of Results	Key points
Robinson [8]	Review/Commentary	Discuss issues and strategies for faculty development related to the use of technology in the classroom	Convincing faculty of the benefits of technology may help mitigate concerns of increased workload. Effective faculty development and training is needed to increase usage.	Investment in resources helps drive the successful implementation of technology. Support for faculty with implementation is crucial.
Kim et al. [9]	Cross-Sectional Survey	Examine the effect of gender and readiness for change on teacher’s self-esteem and technology readiness	Significant correlation between technology readiness and self-esteem (*p* < 0.01). Significant correlation between readiness for change and self-esteem (*p* < 0.01).	Increasing self-esteem and willingness to change improves technology readiness.

**Table 2:** Major diffusion of innovation types and example characteristics. Adapted from Rogers [15], Robinson [8], and the Boston University School of Public Health [16].

Personality Type	Estimated % of Population	Key Characteristics
Innovators	2.5%	Visionary. Risk takers.
Early adopter	13.5%	Embrace change. Key opinion leaders.
Early majority	34%	Need to see evidence before change. Quicker to adopt than the average person.
Late majority	34%	Do not like change. Slower than average to adopt new innovations.
Laggards	16%	Averse to change. Unlikely to introduce or try new innovations.

**Table 3:** Assessment and Recommendations for Each Personality Type.

TechType	Pros	Cons	Recommendations
Expert	Skilled and persistent. Able to solve complex problems.	May exhaust all new ideas and not feel challenged. Possible impatience or inability to relate to slower adopters.	Expand exposure to outside your discipline to learn new information and stay challenged.
Budding Guru	Ambitious and enthusiastic. Potential to serve as a mentor for others.	May require significant support to reach proficiency.	Join or create a user group to share knowledge with others.
Adventurer	Willing to try new ideas. Generates innovative solutions for others.	Tries too many things at once. Takes more risks, which could lead to implementation failures.	Partner with a cautious optimist to balance strengths. Practice before implementation to coordinate logistics and prepare for contingencies.
Cautious Optimist	Guarded approach helps prevent failures. Provides careful consideration and skepticism.	Only partially employs the new tool or feature and therefore may miss its full potential. Restrains creative thinking.	Partner with an expert, budding guru, or adventurer. Beta-test the technology. Consider potential setbacks and brainstorm solutions with others.
Techy Turtle	Consistent approach. Helps prevent the pursuit of technology for the wrong reasons.	Lags behind technology norms. May balk at efforts to standardize processes or change academic space to accommodate innovations.	Work with an IT specialist or budding guru/expert to learn new information and build comfort. Start small and go slow.

## Data Availability

No new data were created in this study. Data sharing is not applicable to this article.
